# Decoding the intercellular communication network during tumorigenesis

**DOI:** 10.20892/j.issn.2095-3941.2021.0558

**Published:** 2021-11-17

**Authors:** Liwei An, Ruixian Yu, Yi Han, Zhaocai Zhou

**Affiliations:** 1 State Key Laboratory of Genetic Engineering, Zhongshan Hospital, School of Life Sciences, Fudan University, Shanghai 200438, China; 2 Department of Medical Ultrasound, Shanghai Tenth People’s Hospital, Tongji University School of Medicine, Shanghai Engineering Research Centre of Ultrasound Diagnosis and Treatment, Shanghai 200072, China

Under physiological conditions, decisions about cell fate and behavior are not made by the individual cell itself, but instead strictly depend on the collective interactions of the whole cell population in the microenvironment. Thus, continuous intercellular communication influences almost all biological processes ranging from tissue/organ development, and homeostasis maintenance, to disease progression. Regarding tumorigenesis, intercellular communications between cancer cells and other cells coexist within microenvironments involving stromal cells, immune cells, and even neural cells, playing crucial roles in tumor initiation, progression, and distal metastasis. Thus, a detailed understanding of cell-cell interactions within the tumor microenvironment facilitates mechanistic delineation of tumorigenesis, and also may identify promising cell-cell interaction targets to treat cancer. One such excellent example is the development of immune checkpoint blockage therapy targeting the PD-1 and PD-L1 pair of ligand-receptor interactions.

Nevertheless, it remains technically challenging to trace transient or cell type specific intercellular interactions and their biological consequences *in vivo*, which illustrates the urgent need to dissect how cell-cell communications influence cancer cell fate, cause tumor heterogeneity, and drive inter-organ metastasis. Fortunately, some elegant tools have been developed in the last 5 years to decode intercellular or even inter-organ communication, which may greatly help us to construct the spatiotemporal cell-cell interaction landscape during tumorigenesis. By summarizing the principles of available intercellular tracing systems, we aim to inspire more in-depth characterization of cell-cell interactions in cancer. Beyond applications of these currently available techniques, we also hope to stimulate more effort in developing next-generation systems that hold promise for tracing cell-cell interactions in a digital manner and/or at a single cell resolution.

## Tracing direct cell-cell interactions

### Synthetic notch receptors (synNotch)

**Figure 1:**
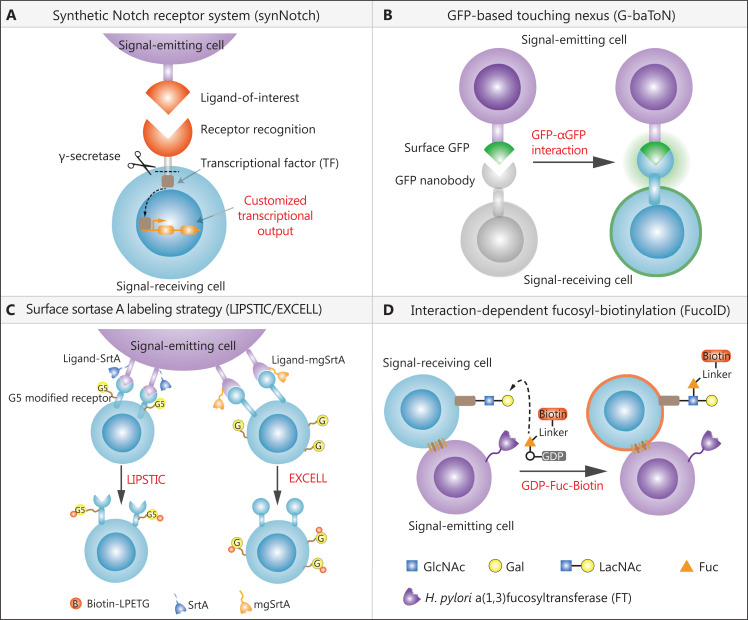
Available techniques used for tracking direct cell-cell interactions. (A) The synNotch system can track interaction between cells with known ligand receptor pairs. The unique advantage of this system is that it can customize cell-cell interactions that trigger intracellular transcriptional outputs. (B) The green fluorescent protein-based touching nexus system is dependent on the transfer of immunofluorescence proteins such as green fluorescent protein from signal-emitting cells to signal-receiving cells upon direct cell-cell interactions. It can also be used to transport functional molecular cargos such as nucleic acids to contacted recipient cells, for modulating cellular responses. (C) Transpeptidase sortase A-dependent LIPSIC requires the genetic manipulation of both ligand and receptor expression cassettes, which prevents it from tracing known cell-cell interactions (left panel). In contrast, mgSrtA, a mutant derived from SrtA-directed evolution, can transfer the substrate to a wide range of cell surface proteins with N-terminal mono-glycine residues without the need for manipulation of receptor molecules, facilitating identification of unknown cell-cell interactions (right panel). (D) Interaction-Dependent Fucosyl-Biotinylation mainly utilizes the glycosyltransferase fucosyltransferase (FT) enzyme to catalyze the fucosyl-biotinylation of surface proteins on contacted nearby cells, facilitating tracking of unknown cell-cell interaction. The unique feature is that the installation of FT on signal-emitting cells is based on chemical self-catalyzation without genetic modification.

The Notch signaling pathway plays multiple roles in the regulation of both the physiology and pathology of various tissues/organs. Inspired by native Notch recognition elements, Lim and co-workers^1^ in 2016 developed a synNotch system to facilitate recording of extracellular ligand receptor interaction-mediated nuclear customized transcriptional outputs. The synNotch receptor contains 3 functional modules: an extracellular receptor, an intact Notch transmembrane core, and a customized transcriptional factor (TF) (**Figure 1A**). Mechanistically, upon trans-engagement of the receptor by an extracellular ligand, the cleavage sites within the transmembrane core are exposed to γ-secretase, a membrane-embedded protease that cleaves a large group of transmembrane proteins. The released intracellular TF domain will then shuttle into the nucleus to initiate customized gene transcriptional activities (**Figure 1A**). Thus, this platform can record defined cell-cell interaction events, and can convert environmental signals (inputs) into customized cellular responses (outputs) in signal-receiving cells. Due to its great advantage for genetic manipulation, the synNotch system has been widely exploited in studying tumor-immune cell interactions, especially for chimeric antigen receptor therapy (SynNotch-CAR)^2,3^. For example, immune cells such as T or NK cells were engineered to stably express synNotch with an antigen-of-interest receptor and customized therapeutic response factors. Thus, the SynNotch-CAR immune cells can selectively kill tumor cells with antigen-of-interest; such a strategy enhances killing efficiency but reduces off-target effects compared with traditional CAR therapy, regardless of tumor specificity and heterogeneity. In addition, the synNotch receptor was also successfully used to visualize direct cell-cell interactions in fly tissue *in vivo*, and to study cell-cell competition in developing epithelia^4^.

Despite multiple elegant examples of the study of cell-cell interactions using the synNotch receptor system, there are currently several limitations preventing wider use of this approach. Due to varying binding affinities between cognate ligands and receptors, the mechanical forces mediated by these ligand-receptor interactions may not always trigger cleavage of the TF domain. Thus, for a defined ligand receptor interaction, extensive trials, optimizations, and validations are required to eventually construct a suitable synNotch system. Moreover, because the native Notch signaling pathway lacks modules for signal amplifications, the sensitivity of synNotch highly depends on the stoichiometry of ligands, which may fail to detect and record low abundance ligand-mediated activation of synNotch receptors. In addition, several groups have reported self-activation of the synNotch receptor even in the absence of ligand. Such leakage of customed transcriptional outputs restrains its potential application *in vivo* for genetic tracing of cell-cell interactions. Overall, further efforts are needed to improve the sensitivity but reduce the self-activation of the synNotch system.

### Green fluorescent protein (GFP)-based touching nexus (G-baToN)

In 2020, Winslow and co-workers developed another tool to label and track direct cell-cell interactions, which was named G-baToN^5^. Briefly, this system utilizes the observations that cell-cell interactions usually induce transendocytosis or trogocytosis-mediated exchanges of surface proteins between contact cells. The design rationale is that signal-emitting cells transfer the surface fluorescent protein (such as GFP) to its interacting signal-receiving cells, which can be further traced using immunofluorescence and/or flow cytometry techniques (**Figure 1B**). The authors showed that this platform could be used in a wide range of cell types such as cancer cells, stromal cells, endothelial cells, neuron cells, and immune cells. More importantly, they showed that the G-baToN system can also be used to transport cargos, such as protein or DNA, to signal-receiving cells and thereby manipulate their cellular behaviors. Despite the simplicity and wide application of this two-component system, so far there has been no *in vivo* evidence to demonstrate its feasibility for intercellular communication analysis under physiological or pathological conditions. Meanwhile, it is highly possible that the molecular transfer may require a relative longer time period, so whether G-baToN can capture transient cell-cell interactions still needs further investigation.

### Proximity-dependent cell surface labeling strategy

In addition to synNoth and G-baToN, an alternative strategy to trace direct cell-cell interactions is based on enzyme-mediated proximal labeling at cell plasma membranes. In 2018, Victora and co-workers^6^ developed a technique named Labeling Immune Partnerships by SorTagging Intercellular Contacts (LIPSTIC) to enzymatically label interacting pairs of cells involved in a specified ligand-receptor interaction. The core of this system harnesses an enzyme named transpeptidase sortase A (SrtA), which is derived from *Staphylococcus aureus*. Functionally, SrtA selectively recognizes neighboring proteins containing 5 successive glycine residues (G5) at its N-terminus, and efficiently transfers substrate polypeptides such as the LPXTG peptide to these residues. Using LIPSTIC, a defined pair of a ligand and receptor is genetically engineered to fuse with SrtA (signal-emitting cell) and G5 oligopeptide tag (signal-receiving cell) (**Figure 1C, left panel**). Upon ligand receptor mediated cell-cell interaction, the SrtA on the signal-emitting cell transfers the biotin-LPETG substrate to covalently label the G5 positive recipient cells (**Figure 1C, left panel**). The key advantage of this technique is that the labeled cells can be sorted and identified even after physical disassociation of the ligand from the receptor, enabling chemical recording of cell-cell interactions. Using this platform, Victora and co-workers^6^ successfully monitored the antigen presentation process between dendritic cells and CD4^+^ T cells using the CD40-CD40L interaction *ex vivo*, and discovered 2 distinct interaction patterns between early and late T-cell priming processes. However, although LIPSTIC can be used with different ligand-receptor pairs, it requires advanced knowledge of the identities of ligands and receptors, which limits the study of intercellular communications mediated by unknown ligands/receptors. To overcome this issue, Chen and co-workers^7^ used a directed evolution method to obtain a SrtA variant (mgSrtA), which tagged the biotin-LPETG substrate to the N-terminal mono-glycine residue. Because certain abundantly expressed endogenous membrane proteins naturally possess an N-terminal mono-glycine residue, there is no need for pre-engineering of signal-receiving cells, thus facilitating detection of intercellular interactions without prior knowledge of the involved cell types. The improved version of this approach was renamed enzyme-mediated proximity cell labeling (EXCELL) (**Figure 1C, right panel**).

In 2020, Wu and co-workers^8^ developed another enzyme-mediated labeling system named Interaction-Dependent Fucosyl-Biotinylation (FucoID) to trace direct cell-cell interactions. The FucoID system takes advantage of the glycosyltransferase fucosyltransferase (FT) enzyme derived from *Helicobacter pylori*. Similar to the role of mgSrtA used in LIPSTIC and EXCELL, the FT enzyme rapidly transfers GDP-fucose (GDP-Fuc) to LacNAc (and/or a 2,3-sialyl LacNAc) residues, which are abundantly expressed on the surface of most cell types (**Figure 1D**). FucoID can potentially trace cell-cell interactions mediated by unknown molecules or even unknown types of recipient cells. To validate this platform, Wu and co-workers added the FT enzyme to the plasma membrane of tumor cells (signal-emitting cells), and successfully identified 3 distinct types of T cells associated with tumor specific antigens within the tumor microenvironment, when combined with RNA sequencing. Further detailed characterization of these 3 T-cell populations revealed novel insights into the tumor sensitivity towards immune checkpoint blockade therapy. A unique advantage of FucoID is that the FT enzyme can be installed onto bait cells using self-catalyzed enzymatic labeling in the presence of GDP-fucose, without the requirement of genetic modification, providing a more feasible method for mapping interactive cells of interest. Nevertheless, compared to the stable expression of the mgSrtA genetic cassette, the abundance of FucoID tethered onto the cell surface will gradually decrease with increasing incubation times. It is estimated that FucoID can only last 10 h, so it faces challenges in the study of slow-dynamic or long-distance cell-cell interactions *in vivo*.

## Tracing indirect cell-cell interactions

Indirect intercellular communication refers to interactions that do not involve direct contact between cells, usually involving secretory proteins and/or extracellular vesicles. During cancer development, it is believed that the establishment of distant metastasis highly depends on indirect cell-cell interactions and even inter-organ communications. To date, several techniques and tool systems have been devised and successfully used to characterize cell-type specific indirect intercellular communications.

### Secreted lipid permeable-mCherry (sLP-mcherry)

**Figure 2:**
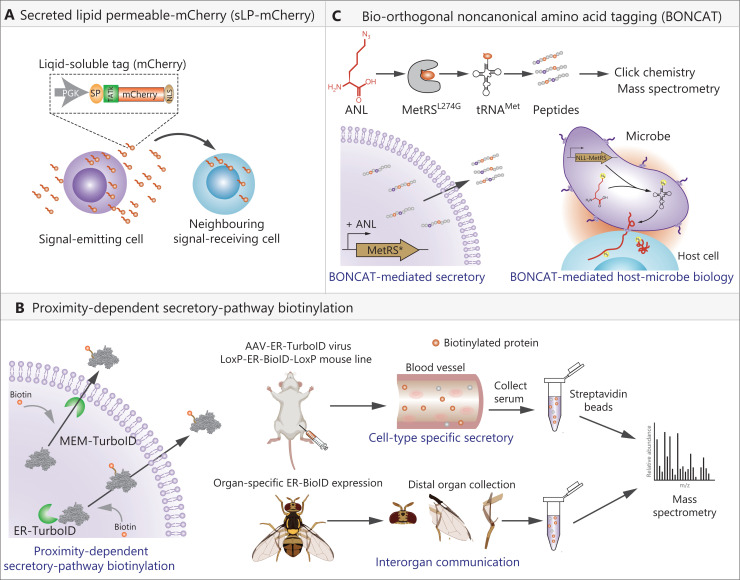
Current techniques used for tracing indirect cell-cell interactions. (A) The sLP-mCherry system is used for studying local interactive cellular composition for a given cell type. The signal-emitting cells expressing a secreted fluorescent protein can be further taken up by nearby signal-receiving cells, facilitating identification of interactive cell compositions. (B) The proximity-dependent secretory-pathway biotinylation strategy relies on engineering the biotin ligase BirA* (or TurboID) to endoplasmic reticulum (ER-TurboID) or plasma membrane (Mem-TurboID), resulting in biotinylation of secreted proteins upon addition of biotin substrate (left panel). This strategy has been successfully used to profile cell type specific secretion in blood plasma (in the mouse model), and for inter-organ communication analysis (in *Drosophila*). (C) Bio-orthogonal noncanonical amino acid tagging facilitates labeling of all freshly synthesized proteins with azide-modified noncanonical amino acids such as azidonorleucine or azidohomoalanine, which can be further detected and enriched using click chemistry. Mammalian cells or microbes stably expressing MetRS* can similarly induce the labeling of secreted proteins as well as those taken up by neighboring cells.

To investigate the cellular composition of the metastatic niche among bulk background tissue *in vivo*, Malanchi and co-workers^9^ developed an mCherry niche labeling system in 2018. Briefly, they fused a secreted fluorescent mCherry protein with a modified lipid-permeable transactivator of the transcription peptide (sLP-mCherry), so that the secreted mCherry released from signal-emitting cells could be taken up by neighboring cells within the microenvironment, providing a system that enabled the identification of local cellular compositions (**Figure 2A**). Using this tool, Malanchi and co-workers characterized a lung epithelial compartment within the metastasis niche originating from breast cancer cells. Due to the relative short half-life of mCherry taken up by neighboring signal-receiving cells (approximately 40 h) and the decreasing levels of mCherry protein along the traveled distance in the circulation and tissues, this system was unable to trace inter-organ communications *in vivo*, especially for inter-organ communications that occurred within a relatively long period of time. While the sLP-mCherry system does help in probing the cell types surrounding signal-emitting cells, it cannot provide detailed molecular characteristics involving intercellular communication. In addition, although secreted mCherry is also found in extracellular vesicles, this form cannot be detected by flow cytometry analysis of neighboring cells, suggesting that the labeling activity of this system is mainly mediated by free secreted mCherry.

### Proximity-dependent secretory-pathway biotinylation

The blood plasma contains almost all the circulating proteins involved in indirect intercellular communications. Multiple types of tissues and cells may contribute to the production of the same secreted protein, and a circulating protein may travel to various organs. In this regard, even though the techniques to identify low abundance proteins have been greatly advanced, it remains challenging to identify the specific tissue/cell origins and possible destinations for a given protein in blood plasma. To address this issue, 4 labs simultaneously and independently used *Escherichia coli*-derived biotin ligase BirA*, which can biotinylate neighboring proteins within an approximate 10 nm range in the presence of biotin, resulting in covalent attachment of biotin to protein substrates^10–13^. BirA* (or its improved version, TurboID) can then be spatially restrained to a defined organelle membrane related to a protein secretion pathway such as that of the endoplasmic reticulum (ER) or plasma membrane (MEM) to undistinguishably biotinylate all secreted proteins, providing a versatile tool for labeling, detection, and enrichment of secreted proteins (**Figure 2B, left panel**). Moreover, once the expression cassette is engineered using a given cell type *in vivo*, the cell-selective secretome can be simply analyzed using serum plasma-based streptavidin enrichment followed by mass spectral analysis (**Figure 2B, right panel**). Furthermore, the secreted proteins taken up by certain tissue/cell-of-interest can also be identified using enrichment-based proteomics, to facilitate the study of secreted protein-mediated inter-organ communication (**Figure 2B, right panel**).

As an example, Long and co-workers^10^ constructed 3 spatially restricted TurboID systems and validated the labeling of the classical ER-Golgi-plasma-dependent secretory pathway by utilization of ER- or MEM-TurboID, as well as validating nonclassical secretions such as through plasma membrane pores or vesicles by cytoplasmic TurboID. By using adeno-associated virus (AAV) to deliver the TurboID system in a mouse model, they also successfully profiled 4 liver cell type-specific secretomes in mouse blood plasma, and tracked the temporally dynamic reprogramming of hepatocyte secretomes following a high fructose and high sucrose diet stimulus. Similarly, Suh and co-workers^11^ used the ER-anchored TurboID and AAV delivery system to profile liver tissue-specific secretory proteins. They named this method *in situ* Secretory protein Labeling *via* ER-anchored TurboID. Finkel and co-workers^12^ further refined the methodology by generating a floxed ER-BioID transgenic mouse, which facilitated delineation of tissue/cell-specific secretion analyses *in vivo*. In addition, Perrimon and co-worders^13^ engineered the ER-BirA*G3, another mutant version of BirA*, into the muscle tissue of *Drosophila*, and successfully detected some labeled secretory polypeptides in the brain, and vice versa, which demonstrated the feasibility of this strategy for tracing inter-organ communication. Collectively, these studies established a proximity-dependent biotinylation strategy to label, detect, and enrich cell type-specific secretomes for the study of interactions between cells and even between tissues/organs.

### Bio-orthogonal noncanonical amino acid tagging (BONCAT)

Given its ability to label newly synthesized polypeptides in specific classes of cells, BONCAT represents another powerful tool for tracking secretory protein-mediated cell-cell interactions. The underlying principle is that cellular translational machinery can recognize and incorporate noncanonical amino acids (ncAAs) into freshly synthesized proteins without perturbation of the metabolic state of the cells. Because the ncAAs are usually pre-modified with azide groups, the synthesized proteins can be further labeled, tracked, and enriched by azide-alkyne cycloaddition (also known as click chemistry) (**Figure 2C, upper panel**). Currently, there are 2 widely used ncAAs, the methionine surrogates, azidohomoalanine (AHA) and azidonorleucine (ANL). AHA can be incorporated by endogenous methionyl-tRNA synthetase (MetRS), while ANL can only be recognized by a mutant version of MetRS (L274G, hereafter referred as MetRS*). On this basis, the generation of a floxed MetRS* mouse line enabled cell-type-specific profiling of nascent proteins *in vivo*^14^.

Tirrell and co-workers^15^ first used BONCAT to characterize intercellular communication between bacteria and their hosts. They co-cultured the engineered *Yersinia enterocolitica*, which stably expressed MetRS*, together with host HeLa cells. Because the newly synthesized bacterial proteins were labeled with an azide group upon addition of ANL, the corresponding secreted proteins including those virulent ones injected into host cells could be distinguished and separated by examination of the culture medium and host cells, respectively (**Figure 2C, lower panel**). Thus, the BONCAT system facilitated selective enrichment of microbial proteins within host cells, to identify new targets for antimicrobial therapy. By using BONCAT in combination with pulse stable isotope labeling with amino acids in cell cultures (SILAC), Leuschner and co-workers^16^ successfully characterized hypoxia-induced secretomes of primary cardiomyocytes. In their experimental setting, the primary murine cardiomyocytes were incubated with AHA to label the freshly synthesized proteins, subsequently subjected to hypoxia; and then the supernatants were collected for proteomics analyses (**Figure 2C, lower panel**). Overall, BONCAT possesses many advantages for analyses of intercellular communications. It can be used within primary cells to label secreted proteins from both classical and nonclassical secretory pathways. Due to the lack of an endogenous blood background, it displays higher sensitivity when compared with the aforementioned biotin-dependent proximity strategy. However, a major limitation of BONCAT is that it cannot label proteins that have existed before addition of methionine surrogates, resulting in the inability to detect information of stored proteins.

## Perspectives

Given the ubiquitous role and central importance of cell-cell interactions *in vivo*, sophisticated techniques have been developed to characterize the elaborate networks that underlie both physiology and pathology, which will increase our understanding of biological processes at the molecular level, and also increase our understanding at the level of cell-cell and even organ-organ communications. Regardless of their optimal applications, these techniques aim to: a) trace and modulate signal-receiving cellular responses upon interaction with signal-emitting cells with known cognate ligands/receptors such as synNotch, G-baToN, and LIPSTIC; b) decipher the unknown cell interactive landscape for a given cell type such as EXCELL, FucoID, and sLP-mCherry; and c) identify the exchanging molecules between signal-emitting and signal-receiving cells/organs such as MEM/ER-TurboID and BONCAT.

Current applications of the summarized techniques remain limited, especially those under physiological and pathological conditions, which are still at their early stages of investigation, with many challenges. Although these systems were all able to trace cell-cell interactions *in vivo*, most validation experiments were conducted under *ex vivo* conditions using well-defined classical and high abundance cell types. Whether these methods can be utilized in disease conditions such as tumor development, to identify novel intercellular networks, still awaits further study. Importantly, the current systems mainly focus on protein-mediated intercellular communications, while minimal studies have involved additional classes of biological molecules such as RNAs and metabolites, which have also been shown to play important roles in cell-cell communications. In this regard, another proximity-dependent enzyme, APEX2, evolved from ascorbate peroxidase of soybeans, has demonstrated a potential capacity to track RNA-mediated intercellular communications. Functionally, APEX2 catalyzes oxidation of biotin-phenol (BP) to produce BP radicals that covalently bind to both neighboring proteins or RNAs^17^. Ideally, by restraining APEX2 expression in certain secretory organelles, it should be possible to label, detect, and enrich secreted RNAs or even RNAs engulfed by recipient cells. However, the use of H_2_O_2_ as a substrate may limit such applications in live, free-moving animals.

With the rapid development of systems dissecting intercellular communications, we see hope for developing a complete and dynamic map of intercellular networks during tumorigenesis. In the future, more advanced technical tools involving proteins, RNAs, and metabolites are required to resolve the specific molecules involved in intercellular communications. In addition, because of their excellent sensitivities and specificities, next-generation techniques are also expected to trace *in vivo* cell-cell interactions in a digital manner and/or at the resolution of single cells, which should quantitatively characterize the critical/individual events involving the initiation, metastasis, and heterogeneity of tumor cells.
